# AL type renal amyloidosis with cardiac involvement: A case report and literature review

**DOI:** 10.1097/MD.0000000000042161

**Published:** 2025-04-18

**Authors:** Yang Sun, Yan-hong Zhao, Qiong-fen Wang, Shan-lan Yang, Yu-juan Yang, Xiao-yu Yang, Ming-wei Liu

**Affiliations:** a Department of Nephrology, Sixth Affiliated Hospital of Kunming Medical University, Yuxi, China; b Department of Gastroenterology, Dali Bai Autonomous Prefecture People’s Hospital, Dali, China; c Department of Oncology, Dali Bai Autonomous Prefecture People’s Hospital, Dali, China; d Department of Nephrology, Dali Bai Autonomous Prefecture People’s Hospital, Dali, China; e Department of Emergency, Dali Bai Autonomous Prefecture People’s Hospital, Dali, China.

**Keywords:** AL type renal amyloidosis, cardiac amyloidosis, case report, diagnosis, treatment

## Abstract

**Rationale:**

Systemic amyloidosis refers to a spectrum of diseases characterized by extracellular deposition of amyloids, with commonrenal involvement; however, simultaneous involvement of cardiac amyloidosis remains rare. The purpose of this report was to enhance the understanding of the diagnosis and treatment of light chain (AL)-type renal amyloidosis with cardiovascular involvement, reduce misdiagnosis and missed diagnosis, and enable timely and effective treatment for such patients.

**Patient concerns:**

A 74-year-old male patient was admitted to our hospital because of recurrent edema for over 1 year and aggravated recurrence with decreased urine for 1 month. Pathological examination of the renal biopsy specimen showed homogeneous nonstructural material deposition in the glomerular mesangial area.

**Diagnoses:**

This patient was diagnosed with lightchain (AL)-type renal amyloidosis with cardiac involvement.

**Interventions:**

Treatment with daretomumab (DARA) 800mg plus cyclophosphamide 0.4g, bortezomib 1.6mg and dexamethasone 20mg (D-VCD regimen). Interventions, such as intermittent diuresis, anticoagulation, and electrolyte imbalance, were administered.

**Outcomes:**

After 2 courses of chemotherapy, edema was relieved, urine protein was reduced, and symptoms improved. Chemotherapy was continued in accordance with the regimen described above.

**Lessons:**

Due to the rarity and nonspecific symptoms, missed diagnosis and misdiagnosis of AL-type renal amyloidosis remain common. Among patients with a confirmed diagnosis, in addition to considering the pathological changes in the kidney, consideration of the presence of amyloidosis in other organs, such as cardiac involvement, is also necessary.

## 1. Introduction

Amyloidosisisa systemic disease characterized by extracellular deposition of amyloids with β-lamellar structures.^[1]^Accumulation of amyloids in tissues or organs can lead to structural destruction and organ dysfunction, which involves multiple organs throughout the body, and renal involvement is a common manifestation of systemic amyloidosis.^[1]^More than 30 amyloid substances are known, and precursor proteins that can cause renal involvement include different types such as amyloid light chain (AL), AA, Aβ2m, AFib, AApoAI, AApoAII, and ALys. AL amyloidosisaccounts for 70% to 80% of all types of amyloidosis^[2]^ with immunoglobulin light chain as the precursor protein. Although AL amyloidosis is a rare disease, its prevalence in the United States has been reported to increase significantly from 15.5 per million population (PMP) in 2007 to 40.5 PMP in 2015, with an annual rate of change of 12%, and the incidence fluctuates from 9.7 to 14.0 PMP.^[3]^ While in the United Kingdom, a total of 11,006 amyloidosis cases were diagnosed from 1987 to 2019, and the number of cases increased by 670% from to 1987–1999 to 2010–2019, with AL amyloidosis accounting for 55%.^[4]^

AL-type renal amyloidosis has rarely been reported in China,^[5]^ especially with comorbid cardiac involvement. As nonspecific symptoms of renal amyloidosis with cardiac involvement, missed diagnosis and misdiagnosis can easily occur, resulting in delayed treatment and adverse prognosis. In this study, we report the diagnosis and treatment of renal amyloidosis with cardiac involvement to shed light on clinical reference and avoid missed diagnosis and misdiagnosis in the future.

## 2. Case presentation

### 2.1. Ethics approval and consent to participate

Informed written consent was obtained from the patient and his daughter for the publication of this case report and accompanying images. This study was reviewed and approved by the local ethics committee of the Sixth Affiliated Hospital of the Kunming Medical University. The procedures were in accordance with the Helsinki Declaration of 1975, as revised in 2000.

### 2.2. Medical history

A 74-year-old male patient was admitted to the nephrology department of the People’s Hospital of Yuxi City in June 2022 due tolower extremity edema without predisposing causes for over 1 year, occasional eyelid edema, 4 to 5 times of daytime urine, one time of night urine, approximately 150 to 200 mL urine volume each time, and foamy. The albuminuria/creatine ratio was 175.65 mg/mmol, 24-hour urinary protein quantity was 1945.6 mg. A diagnosis of diabetic nephropathy was made, and renal biopsy was rejected. The patient received kidney protection, urine protein lowering, and other treatments and was discharged with relieved edema. Regular outpatient follow-up examination showed that urinary protein gradually increased, 24-hour urinary protein fluctuated between 3 and 5 g, and edema was mild and sometimes severe. In the last month, edema recurred and aggravated, with a daily urine volume of approximately 500 mL, and no urination discomfort, palpitation, chest tightness, rash, joint pain, or fever were reported. On February 13, 2023, the patient was readmitted to the nephrology department of the People’s Hospital of Yuxi City.

### 2.3. Personal history

The patient had elevated blood pressure for 3 years, with the highest blood pressure of 230/160 mm Hg. He recently took amlodipine mesylate 10 mg once daily (QD), and the pressure fluctuated between 30 to 200/90 to 110 mm Hg. There was no history of diabetes, cardiovascular or cerebrovascular disease, or other important organ diseases such as lung, kidney, orendocrine system diseases. There was no history of infectious disease, trauma, surgery, blood transfusion, or allergy. The vaccination regimen was unknown.

### 2.4. Physical examination

Body temperature, 36.5°C; pulse, 73 beats/min, respiration 18 times/min, blood pressure 104/77 mm Hg, body height, 166 cm; weight, 80 kg; body mass index, BMI: 19.3 kg/m^2^. Clear consciousness, no pale on skin and mucosa; slightly swollen face and eyelids; no distention of bilateral jugular veins; breath sounds in both lungs were coarse, without dry or moist rales or pleural friction rub. No bulge or depression in the precordium, the apical pulse point was 0.5 cm in the left midclavicular line of the fifth intercostal space, normal heart border; heart rate was 73 beats/min, regular rhythm, no murmur or additional heart sounds, no pericardial friction rub. The abdomen was flat and soft, without tenderness, rebound tenderness, or muscle tension. The liver and spleen were not palpable or enlarged, and there was no tenderness at any ureteral point. The lower limbs showed mild pitting edema.

### 2.5. Laboratory results

Routine blood tests on January 18, 2023: white blood cellcount 6.14 × 10^9^/L; neutrophil ratio, 59.2%; hemoglobin, 158 g/L; and blood platelet, 114 × 10^9^/L. Liver function: total protein 56.5 g/L↓, albumin 31.9 g/L↓, globulin 24.6 g/L, albumin/globulin ratio 1.3, alkalinephosphatase 85 U/L, γ-glutamyl transferase 100 U/L↑. Kidney function: urea 5.2 mmol/L, creatinine 89 µmol/L, uric acid 433 µmol/L↑, cystatin 1.29 mg/L. Cardiac biomarkerscreatine kinase 52 U/L, creatine kinase-MB 1.57 µg/L, α hydroxybutyrate dehydrogenase 133 U/L, high-sensitivity cardiac troponin T (hs-TnT)0.029 µg/L↑, myoglobin 63 µg/L, NT-proBNP 568.6 pg/mL↑. Blood lipids: total cholesterol 5.24 mmol/L ↑, triglycerides 1.12 mmol/L, high-density lipoprotein cholesterol 1.61 mmol/L, low-density lipoprotein cholesterol 3.34 mmol/L, very low-density lipoprotein cholesterol 0.49 mmol/L. Blood glucose: fasting blood glucose 4.91 mmol/L, glycated hemoglobin A1c, 5.9%. Humoral immunity: immunoglobulin G 10.76 g/L, immunoglobulin A 1.9 g/L, immunoglobulin M 0.86 g/L, complement C3 0.94 g/L, complement C4 0.26 g/L, complement C1q 0.17 g/L. Electrolytes: potassium 3.97 mmol/L, sodium 142 mmol/L, chlorine 109 mmol/L, total calcium 2.21 mmol/L, phosphorus 1.16 mmol/L, bicarbonate 21 mmol/L. Urine routine: white blood cells (−), urinary protein 3+, occult blood (−), pH 6.5, red blood cells 9/µL, white blood cells 4/µL, crystals 36/µL, β2-microglobulin 0.75 mg/L↑, immunoglobulin > 200.00 mg/L↑, urinary microalbumin > 500.00 mg/L. 24-hour urinary protein: 4740.1 mg/24h↑. Antinuclear antibody spectrum: antinuclear antibody (+), antinuclear antibody titer S + 1:320, others negative. Immune vasculitis: negative for anti-neutrophil cytoplasmic antibodies type C and type P. Infectious immune markers: hepatitis B virus surface antibody 40.03 mIU/mL↑, negative for hepatitis C, syphilis, and HIV. Calcium and phosphorus metabolism: parathyroid hormone 22.52 ng/L, calcitonin 3.94 ng/L. Thyroid function: triiodothyronine 0.51 µg/L↓, tetraiodothyronine 46.24 µg/L↓; hypersensitive thyroid stimulating hormone 12.27 mIU/L↑, serum thyroid binding globulin 65.9 µg/L↑. Comprehensive tumor markers: alpha fetoprotein 9.33 µg/L↑, carbohydrate antigen (CA-199) 26.45 KU/L↑, others were negative. Prostate tumor markers: total prostate-specific antigen 0.75 µg/L, free prostate-specific antigen 0.18 µg/L, FPSA/PSA 0.24. Anti phospholipase A2 receptor antibody was negative. Respiratory system tumor markers were negative.

### 2.6. Renal biopsy and immunofluorescence results

**Figure 1.**
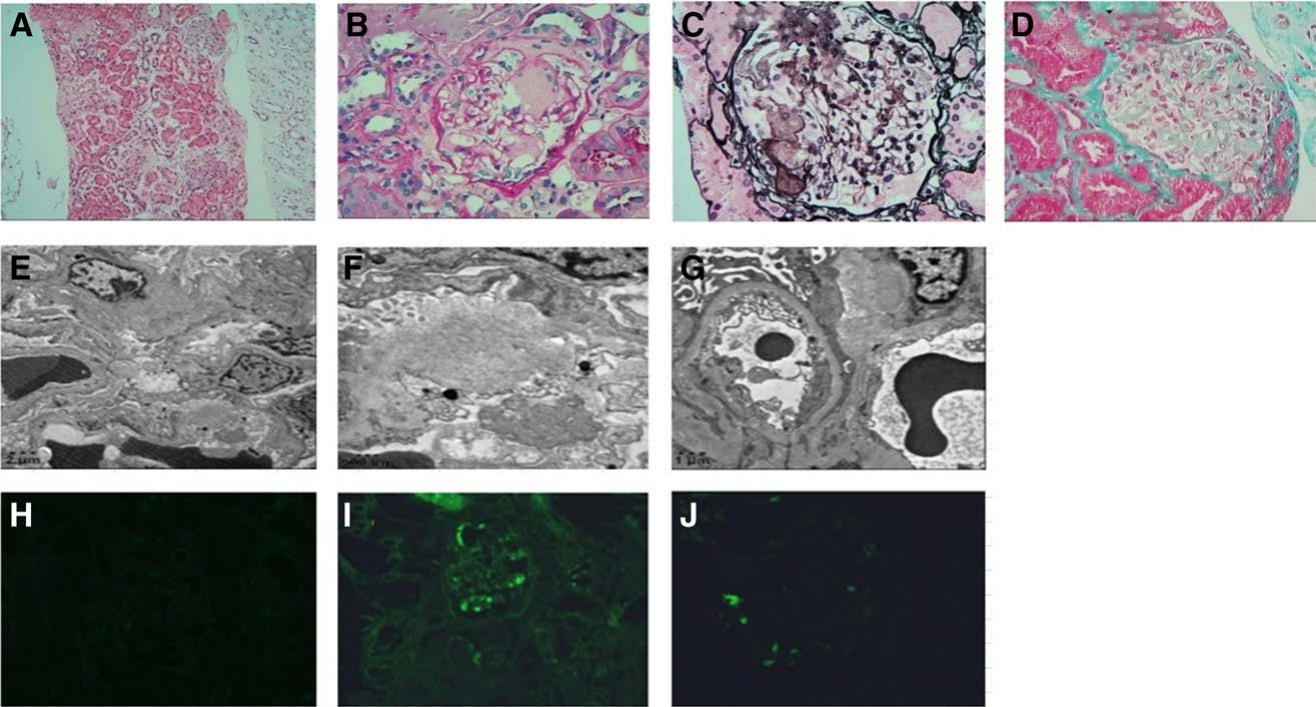
Changes of renal biopsy and immunofluorescence. (A–D) Changes of renal tissue under light microscopy; A: hematoxylin and eosin staining (×100); B: periodic acid-Schiff staining (×400); C: periodic Schiff-methenamine staining (×400); D: Masson staining (×400). (E–G) Ultrastructure of renal tissue. (H–J) Changes of immunofluorescence in renal tissue; H: kappa (−); I: lambda (+); J: IgM (+/−).

Light microscopy: HE, PAS, PASM, and Masson staining were performed routinely in renal biopsy tissues, including the renal cortex and medulla (Fig. 1A–D). A total of 13 glomeruli were observed, including 8 glomerulosclerosis in a relatively concentrated location (Fig. 1A–D). The remaining glomerular mesangial areas had deposition ofstained homogeneous nonstructural material, which widened some glomerular mesangial areas (Fig. 1A–D). Segmental eyelash-like changes were found in the glomerular basement membrane, with compression of the capillary loops (Fig. 1A–D). Vacuoles and granular degeneration were observed in tubular epithelial cells, while infiltration with mild fibrosis was detected in renal interstitial small focal inflammatory cells, arteriolar wall thickening and luminal narrowing, and stained protein-like material deposition was seen (Fig. 1A–D). Electronic microscopy revealed segmental thickening of the glomerular basement membrane and fusion of most foot processes (Fig. 1E–G). There was no electron dense deposition, but massive fibroid deoisutuib in arterioles, mesangial areas, and basement membranes, approximately 8-12 nm in diameter, stiff without branches, and disorganized (Fig. 1E–G). Immunofluorescence: amyloids; kappa (−); lambda (+); AA (−) (Fig. 1H–J).

### 2.7. Bone marrow smear, biopsy and immunohistochemistry

The myelogram showed active proliferation of nucleated cells, mild maturation disorders, and degenerative changes in the granulocytic series (Fig. 1A and B). Immunohistochemistry revealed plasmacytosis (approximately 5%) (Fig. 1A and B).

### 2.8. Imaging results

**Figure 2.**
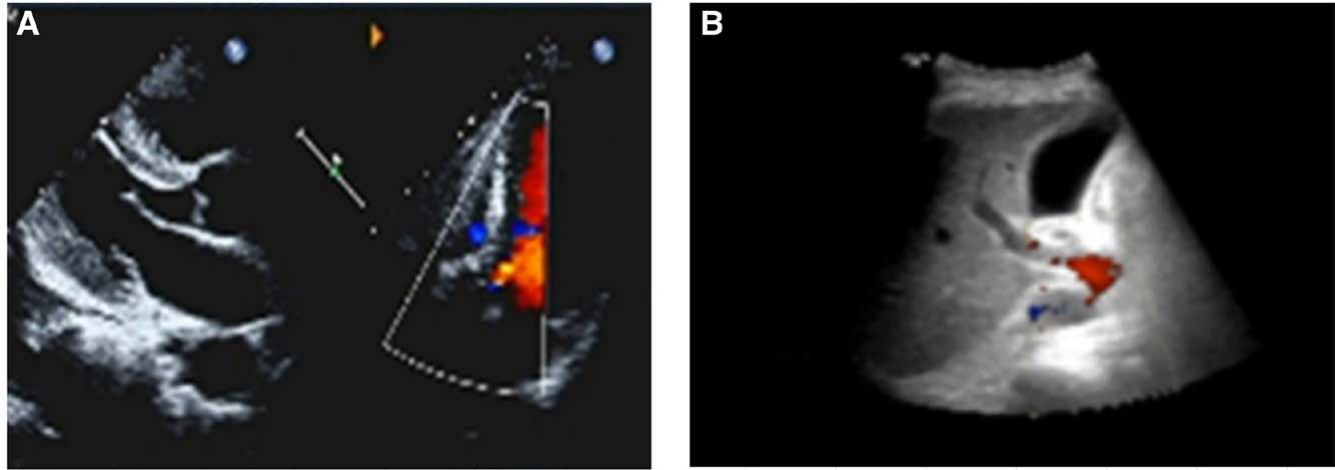
Changes in cardiac ultrasound and abdominal ultrasound during patient admission. (A) Changes of Doppler echocardiography at admission. (B) Changes of abdominal ultrasound at admission.

**Figure 3.**
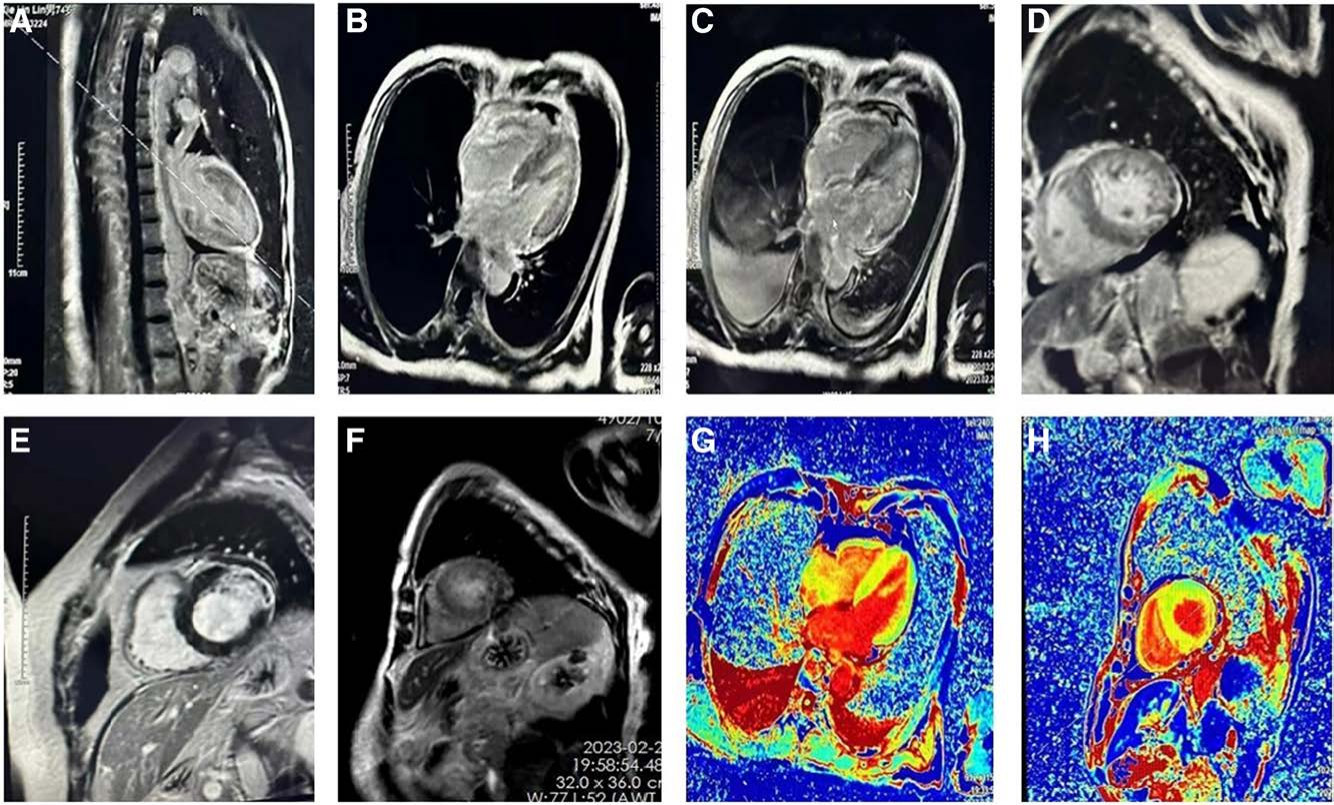
(A–H) Changes of cardiac magnetic resonance imaging at admission.

Abdominal ultrasound revealed multiple cysts in both kidneys and bilateral renal calculi (multiple cysts in the right kidney) (Fig. 2B). Echocardiography: aortic valve calcification with a small amount of regurgitation, ejection fraction of 61%and a small amount of pericardial effusion (Fig. 2A). Cardiac MRI: diffuse delayed enhancement of the left and right ventricular intramural, subendocardial, and interatrial septum, significantly increased native T1 and ECV values of the left ventricular myocardium, slightly thickened interventricular septum, and slightly larger left atrium (Fig. 3A–H). No ischemic cardiomyopathy or acute myocarditis was observed, and no abnormalitiesin cardiac function were observed (Fig. 3A–H). Myocardial amyloidosis was considered based on the patient’s medical history.

### 2.9. Diagnosis and treatments

**Figure 4.**
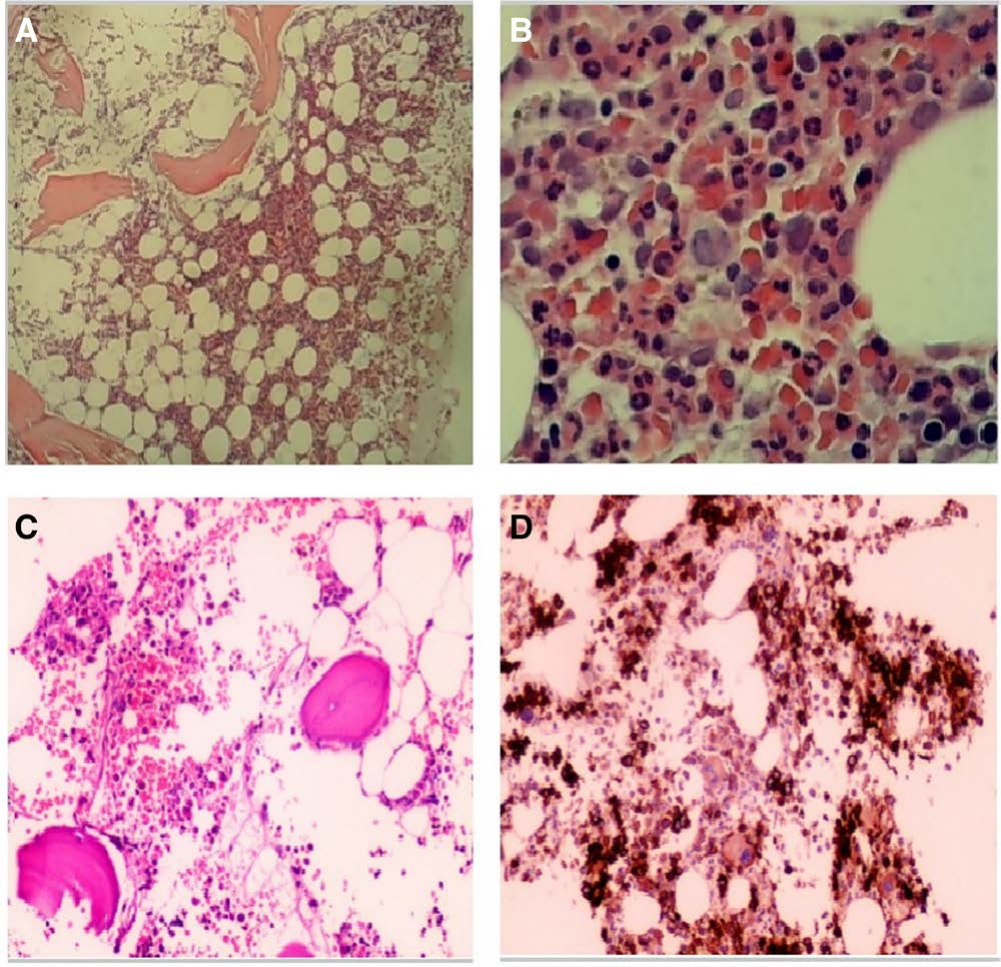
Changes of bone marrow smear, biopsy, and immunohistochemistry. (A and B) Bone marrow smear, biopsy, and immunohistochemistry before treatments. (C and D) Bone marrow smear, biopsy, and immunohistochemistry after treatments.

The patient was diagnosed with AL-type renal amyloidosis with cardiac involvement based on medical history, symptoms, cardiac MRI, and renal biopsy. After admission, he was administered lenalidomide (25 mg QD, orally, for 21 days as a course of treatment) + dexamethasone (15 mg QD, orally, for day 1–4 and 15–18) + chemotherapy. In addition, fenelidone and dapagliflozin were administered ashypoglycemic and urinary protein-lowering treatments. The patient had recurrent episodes of hypotension and syncope 3 times outside the hospital. From April 4, 2023, the treatment was changed to daratumumab (DARA) 800 mg plus cyclophosphamide (400 mg), bortezomib (1.6 mg), and dexamethasone (20 mg) (D-VCD regimen) once a week for 29 days. The first 2 courses were once a week, the third to sixth courses were once every 2 weeks, and the seventh course was once every 4 weeks. After 14 courses of treatment, the edema and general condition improved. The daily urine volume was 1000 to 1200 mL with no syncopal attacks. Blood glucose control was stable, blood pressure fluctuated between 99–110/62–72 mm Hg. The 24-hour urinary protein quantity gradually decreased, while serum albumin gradually increased. The NT-ProBNP, serum creatinine, and hs-TnT levels did not increase significantly (Table 1). Bone marrow smear cytology results showed that nucleated cells accounted for approximately 55%, the proportions of granulocytes and erythrocytes were normal, granulocytic hyperplasia, mainly myelocytes, metamyelocytes, and below; erythroid hyperplasia, mainly polychromatophilic and orthochromatic erythroblasts; megakaryocytes 2 to 4/HPF, no significant abnormalities in morphology; scattered plasma cells were observed in the interstitium (proportion less than 1%) (Fig. 4C and D). Bone marrow biopsy and immunohistochemistry detected 0.01% plasma cells without significant clonality (Fig. 4C and D).

**Table 1:** Changes of 24-hour urinary protein quantity, NT-ProBNP, hs-TnT, creatinine, and serum albumin

Before and after treatment with DARA as the main regimen	Measurement time	24-hour urinary protein quantification (mg/24 h)	NT-proBNP in blood (pg/mL)	hs-TnT in blood (µg/L)	Creatinine in blood (µmol/L)	Serum albumin (g/L)
Before treatment with DARA as the main regimen	June 11, 2022	1946	568	0.029	93	34.0
	February 15, 2023	4740	1626	0.058	90	31.6
	February 26, 2023	5530	3280	0.136	98	21.5
After treatment with DARA as the main regimen (Treatment with DARA starts from April 04, 2023)	April 10, 2023	3896	8526	0.15	118	17.6
	May 10, 2023	4885	2105	0.076	96	18.9
	June 3, 2023	6726	2298	0.079	93	23.7
	June 28, 2023	7530	1744	0.059	84	23.6
	July 25, 2023	5367	1464	0.051	77	27.3
	August 13, 2023	6606	1119	0.041	79	30.1
	September 03, 2023	3858	964	0.034	74	23.8
	October 08, 2023	3524	1192	0.034	79	25.9
	November 25, 2023	3254	777	0.032	87	31.3
	December 30, 2023	2739	699	0.026	87	33.2
	January 27, 2024	3116	1050	0.027	86	33.0
	March 03, 2024	2156	822	0.026	93	33.1
	April 15, 2024	1774	705	0.022	95	33.2
	May 27, 2024	1575	538	0.021	79	27.7
	July 01, 2024	1574	1032	0.019	90	35.9
	August 04, 2024	989	829	0.022	91	37.7
	September 02, 2024	1236	678	0.020	98	33.8
	October 23, 2024	868	705	0.018	89	36.5

D-VCD regimen started on April 04, 2023.

### 2.10. Follow-up after treatments

After regular treatment for half a year, the edema of the lower extremity significantly improved, urine protein decreased (Table 1), and no complaints of discomfort were reported. The patient was regularly admitted to the hospital to continue chemotherapy.

## 3. Discussion

The kidneys are one of the most frequently affected organs.^[6]^ Patients with AL renal amyloidosis mainly present with proteinuria and nephrotic syndrome and further renal insufficiency with disease progression, which seriously affects their quality of life. Due to the lack of characteristic clinical manifestations, the missed diagnosis and misdiagnosis rates remain high in clinical practice, and the diagnosis depends on renal biopsy. A single-center retrospective study in China reported that 98.4% of all AL systemic amyloidosis cases with renal involvement were confirmed via biopsy.^[7]^ Onset age of onset of AL renal amyloidosis is usually ≥ 40 years. Quock et al^[8]^ found that the prevalenceincreased with age; specifically, compared with 35 to 54 years, theprevalence doubled among people aged 65 years and above. The mean age at diagnosis was 63 years, and males were more vulnerable. The patient in this study was a 74-year-old male, consistent with the general age of onset.

AL amyloidosisis a systemic disease with diverse clinical manifestations without specificity; therefore, itsdiagnosis is a major challenge. Although macroglossia and skin purpura are 2 important signs, only 5% and 8% of 153 patients reported such symptoms, respectively, in a previous study by Gertz et al.^[9]^ The early diagnosis rate of this disease is relatively low. For patients over 40 years of age, typical nephrotic syndrome may occur with low blood pressure accompanied by hepatosplenomegaly and multiple system damage. Monoclonal immunoglobulin was detected during examination. Even in patients with end-stage renal disease, there was no significant reduction in kidney volume, suggesting the possibility of amyloidosis nephropathy.^[10]^ The first symptom of our patient was edema of both lower limbs, followed by asymptomatic proteinuria, chest tightness, and shortness of breath after activity, without macroglossia or periorbital purpura. Treatments for nephrotic syndrome and cardiac dysfunction were administered, but with poor therapeutic effect, AL renal amyloidosis was confirmed according to the subsequent kidney biopsy. In recent years, the incidence of AL renal amyloidosis has increased annually, particularly among younger people. Yao et al^[11]^ identified 12 (6.5%) AL renal amyloidosis cases inpatients under 40 years of age. Therefore, immunofixation electrophoresis is also suggested in patients > 30 years of age, and Congo red staining should be routinely performed in renal pathological specimens to expand the screening range for renal damage in amyloidosis.

Microscopic hematuria was found in the patient in this study, accompanied by nonselective proteinuria (mainly albumin) in proteinuria profile. Cardiac ultrasound showed no obvious abnormality, but the NT-ProBNP level was significantly elevated, suggesting the presence of cardiac involvement at admission, which was a high-risk factor for mortality. Cardiac MRI indicated diffuse delayed enhancement of the left and right ventricular intramural spaces, as well as the subendocardium and interatrial septum. In addition, native T1 and ECV values of the left ventricular myocardium obviously increased, the interventricular septum was slightly thickened, and a slightly larger left atrium was found. Myocardial amyloidosis was considered based on the patient’s medical history. Late gadolinium enhancement. The mortality rate is approximately 50% among patients with cardiac involvement within 6 months of diagnosis.^[12]^ The typical LGE pattern of AL amyloidosis is transmural and subendocardial delayed enhancement and is a predictor of poor prognosis for cardiacamyloidosis. The degree of cardiac involvement has an important impact on prognosis, especially for AL amyloidosis combined with multiple organ injury, and the prognosis is poor.

The diagnosis of AL renal amyloidosis mainly relies on renal biopsy with a sensitivity of 100%, whereas the sensitivity of Congo red staining and polarized light examination of subcutaneous liposuction biopsy is 57% to 85%, witha specificity is 92% to 100%.^[13]^ Because of the poor therapeutic effect on nephrotic syndrome at admission, renal biopsy was performed and revealed deposition of stained homogeneous nonstructural material, which widened some glomerular mesangial areas. Segmental eyelash-like changes were found in the glomerular basement membrane, with compression of the capillary loops. Vacuoles and granular degeneration were observed in tubular epithelial cells, whereas infiltration with mild fibrosis was detected in renal interstitial small focal inflammatory cells, arteriolar wall thickening and luminal narrowing, and stained protein-like material deposition was observed. Further immunofluorescence results: IgG (−); IgA (−)；IgM (±)；C3 (−)；C1q (−)；Fib (−)；ALB (−)；kappa (−)；lambda (+)；AA (−)；IgG1 (−)；IgG2 (−)；IgG3 (−)；IgG4 (−)；PLA2R (−)；THSD7A (−). The patient was diagnosed with AL-type renal amyloidosis with cardiac involvement.

AL-type amyloidosisis inherently understood as a complication of plasma cell dysplasia, and its chemotherapy modalities are mostly based on multiple myeloma chemotherapies^[14]^ which rapidly eliminate light chains by targeted treatment of plasma cells, thereby inhibiting the production of precursor proteins.^[15]^ Current studies have proven the satisfactory effectiveness and safety of DARA in treating AL-type amyloidosis.^[16,17]^ A muti-center randomized controlled study on 338 primaryAL amyloidosis patients noted that compared with the cyclophosphamide + bortezomib + dexamethasone group (18.1%), patients receiving DARA hada significantly higherhematologic response(53.3%), better cardiac and renal responses at 6 months of treatment, and better survival prognosis.^[18]^ The above study suggests that the addition of daratumumab (D-VCD) is associated with higher rates of complete hematologic response and survival without major organ deterioration or hematologic progression in newly diagnosed AL amyloidosis. In refractory and recurrent AL amyloidosis with cardiac involvement, DARA can rapidly induce hematological remission and thus prevent further organ involvement, such as the heart.^[19]^ Therefore, after diagnosis of AL renal amyloidosis with cardiac involvement, this patient underwent D-VCD biweekly for nearly one year, after which edema of the lower extremities was obviously relieved and urinary protein decreased. Chemotherapy was continued in accordance with the current protocol.

### 3.1. Strengths and limitations

#### 3.1.1. Strengths

AL-type renal amyloidosis with cardiac involvement is rare, and treatment based on daretomumab has been approved to be effective.

#### 3.1.2. Limitations

Due to its rarity and nonspecific symptoms, missed diagnosis and misdiagnosis of AL-type renal amyloidosis remain common. During the administration of daretomumab and bortezomib, lymphocyte depletion and infection have occurred, and daretomumab is prone to infusion reactions.^[20,21]^ Therefore, blood cell changes and changes in the patient’s condition should be closely monitored during treatment. In addition, different patients show varying responses to immunotherapy, and personalized treatment may be necessary when necessary. Amyloidosis affected the heart, and no pathological examination of the cardiac tissue was performed. However, if the patient’s outcome is poor or the condition worsens, cardiac pathology examination is still necessary. Currently, there is still verylittle research on chemotherapy treatment using daretomumab; multi-center randomized controlled trials with larger sample sizes are needed to further confirm our results. In addition, the exact mechanism underlying AL-type renal amyloidosis remains unclear.

## 4. Conclusion

This case report suggests that AL-type renal amyloidosis is prone to heart involvement. For patients with AL-type renal amyloidosis involving the heart, daretomumab treatment is effective. During the treatment process, close observation of routine blood tests, renal function, hematuria protein, NT-ProBNP, and changes in the condition should be conducted. Due toitsrarity and nonspecific symptoms, missed diagnosis and misdiagnosis of AL-type renal amyloidosis remain common. For patients with comorbid edema of both lower limbs and massive proteinuria, an early renal biopsy is required to make a clear diagnosis. Among patients with a confirmed diagnosis, in addition to considering the pathological changes in the kidney, considerations of the presence of amyloidosis in other organs are also necessary, such as cardiac involvement; thus, cardiac MRI or biopsy should be performed as early as possible.

## Author contributions

**Conceptualization:** Yang Sun, Qiong-fen Wang, Ming-wei Liu.

**Data curation:** Yang Sun, Qiong-fen Wang, Shan-lan Yang, Ming-wei Liu.

**Formal analysis:** Yan-hong Zhao, Shan-lan Yang, Yu-juan Yang, Xiao-yu Yang.

**Funding acquisition:** Yang Sun, Ming-wei Liu.

**Investigation:** Yang Sun, Yan-hong Zhao, Qiong-fen Wang, Yu-juan Yang, Xiao-yu Yang, Ming-wei Liu.

**Methodology:** Yan-hong Zhao, Qiong-fen Wang, Shan-lan Yang, Yu-juan Yang.

**Project administration:** Yang Sun, Qiong-fen Wang, Shan-lan Yang, Xiao-yu Yang.

**Resources:** Yang Sun, Yan-hong Zhao, Shan-lan Yang, Yu-juan Yang, Xiao-yu Yang, Ming-wei Liu.

**Software:** Yang Sun, Yan-hong Zhao, Qiong-fen Wang, Yu-juan Yang.

**Supervision:** Shan-lan Yang, Xiao-yu Yang, Ming-wei Liu.

**Validation:** Yang Sun, Yan-hong Zhao, Ming-wei Liu.

**Visualization:** Yan-hong Zhao, Shan-lan Yang, Yu-juan Yang, Xiao-yu Yang, Ming-wei Liu.

**Writing – original draft:** Ming-wei Liu.

**Writing – review & editing:** Ming-wei Liu.
